# The Impact of Nirsevimab on the Transport of Critically Ill Children

**DOI:** 10.3390/children13020268

**Published:** 2026-02-14

**Authors:** Carme Alejandre, Enrique Pazos, Pablo Gonzalez-Alvarez, Mònica Girona-Alarcón, Nuria Millán, Manuel Rodriguez, Aina Covas, Aina Martinez Planas, Elisabeth Esteban

**Affiliations:** 1 Pediatric Transport Team, Hospital Sant Joan de Déu, 08950 Barcelona, Spain; enrique.pazos@sjd.es (E.P.); pablo.gonzalez@sjd.es (P.G.-A.); monica.girona@sjd.es (M.G.-A.); nuria.millan@sjd.es (N.M.); manuel.rodriguez@sjd.es (M.R.); acovas.girona.ics@gencat.cat (A.C.); aina.martinez@sjd.es (A.M.P.); elisabeth.esteban@sjd.es (E.E.); 2 Pediatric Intensive Care Unit, Hospital Sant Joan de Déu, 08950 Barcelona, Spain; 3 Immunological and Respiratory Disorders in the Pediatric Critical Patient Research Group, Institut de Recerca Sant Joan de Déu, University of Barcelona, 08950 Barcelona, Spain

**Keywords:** bronchiolitis, nirsevimab, monoclonal antibody, transport

## Abstract

**Highlights:**

**What is known about this subject?**

- Respiratory syncytial virus (RSV)-positive bronchiolitis is the leading cause of interfacility transport in children <1 year, especially in winter.
- In October 2023, universal immunization against RSV in children under 6 months of age was started in Spain.

**What does this study add?**

- Immunization with nirsevimab was associated with lower rates of bronchiolitis requiring specialized transport in pediatric patients, particularly in RSV-related cases, without modifying disease severity among those requiring transfer.

**Abstract:**

**Purpose:** Respiratory syncytial virus-positive bronchiolitis continues to be the main diagnosis prompting transportation in children younger than one year of age. It represents approximately 15–20% of all services performed by a specialized pediatric transport team. In October 2023, an immunization program with nirsevimab, a monoclonal antibody against RSV, was started in Spain. The purpose of the present study is to describe how nirsevimab affects the rates of bronchiolitis managed by a pediatric team specialized in critical patient transport. Secondary objectives included describing and comparing the clinical aspects of the two cohorts—pre-nirsevimab (pre-n) and post-nirsevimab (post-n)—to quantify how immunization has modified the clinical phenotype of bronchiolitis. **Methods:** This is a descriptive and observational study. Patients with bronchiolitis transported by a specialized pediatric transport team between September 2021 and August 2025 were included. Demographic, clinical, and microbiological data were collected. The pre-n and post-n periods were compared. **Results:** From a total of 2347 interfacility transports conducted by the unit between 2021 and 2025, 463 (19.7%) involved bronchiolitis patients, all of whom were recruited: 307 in the pre-n period and 156 in the post-n. The median age was 2.5 months (IQR 1.3–5.7), and 55% were male. There was a significant decrease in bronchiolitis cases that required specialized transport between the two periods: 28.2% (307/1089) pre-n vs. 12.4% (156/1258) post-n (*p* < 0.001). RSV detection also declined (74.3% vs. 47.4%, *p* < 0.001), while other viruses increased significantly in the post-n period, including rhinovirus, metapneumovirus and bocavirus. Age at admission showed statistically significant differences across the two periods (2.2 vs. 3.4 months, *p* < 0.001). There were no differences in severity between the two periods in terms of respiratory and inotropic support and length of stay. No mortality was reported. **Conclusions:** Universal nirsevimab immunization was associated with a marked reduction in pediatric transports for bronchiolitis, particularly RSV-related cases, without modifying disease severity among those requiring transfer.

## 1. Introduction

Bronchiolitis is the most common respiratory infection in children, causing significant morbidity and placing a substantial burden on healthcare systems [1,2].

Although most patients can be managed in the primary care setting, a minority of them are eventually admitted to the hospital, representing 18% of all hospital admissions among children under one year of age [3,4]. Between 2% and 6% of the hospitalized patients eventually require intensive care for respiratory support, representing 13% of pediatric intensive care unit (PICU) admissions [5,6,7].

PICUs in Catalonia are located in major tertiary hospitals, generally in the main cities of the region. Management of patients in highly specialized centers has demonstrated better outcomes and is cost-effective [8,9,10]. This centralization creates the need for interhospital transfers of critically ill patients from less complex centers [11,12]. Thirty years ago, specific pediatric and neonatal transport units were established within the Emergency Medical System (EMS), responsible for the majority of interhospital transfers for these critically ill patients. The transport team consists of a pediatrician, a nurse, and an emergency medical technician, with proven expertise in managing critically ill patients. The team transports approximately 600–700 pediatric patients each year, coordinated by a pediatrician at the emergency medical coordination center. The transport of pediatric patients, particularly infants, is closely linked to the epidemiology of bronchiolitis, which is still the leading cause of transfers in children under one year of age. Bronchiolitis caused by RSV leads to predictable seasonal increases in the demand for intensive care capacity and healthcare resources [13,14], leading to frequent transfers from lower-complexity hospitals to tertiary centers with more advanced therapy [15]. There have been no changes in referral policies or patient referral criteria in recent years.

Until recently, the only available preventive agent against RSV was palivizumab, a monoclonal antibody administered monthly to premature babies and patients with chronic diseases [16]. In Spain, maternal vaccination is not administered.

Nirsevimab was approved on 31 October 2022 by the European Medicines Agency (EMA) as a preventive therapy for RSV-related bronchiolitis. Nirsevimab (Beyfortus^®^, AstraZeneca, marketed in Spain by Sanofi Pasteur) is a monoclonal antibody targeting the viral fusion (F) protein, which has demonstrated a favorable safety and efficacy profile in various clinical trials. Notably, a single dose of nirsevimab provides protection for several months, covering the entire RSV season [17,18,19,20,21,22].

Later, on 25 July 2023, the Public Health Commission of the Spanish Interterritorial Council of the National Health System issued the document “Recommendations for the use of nirsevimab against respiratory syncytial virus for the 2023–2024 season”, proposing its universal use in infants under six months of age at the beginning and during the RSV season and in children at high risk for severe RSV disease [23]. Starting in October 2023, the immunization program achieved a coverage of 83,4% of the candidate population [24]. These same recommendations were issued for the following season, achieving similar coverage rates [25].

The introduction of nirsevimab represents a significant advance in reducing the burden of severe bronchiolitis. Multicenter studies in Spain and across Europe have shown how nirsevimab diminishes hospitalizations by more than 60%, cases presenting to the emergency room by 41.6%, and PICU admissions by up to 81%, while also reducing the overall hospital length of stay and the need for advanced ventilatory support [26,27,28,29,30,31,32,33,34,35,36,37,38,39,40,41].

However, despite the significant reduction in hospitalizations and PICU admissions for RSV-related respiratory infections in infants [26,27,41,42,43,44,45,46,47,48], no studies have directly assessed its impact on pediatric transport.

Therefore, the aim of this study was to evaluate the impact of the immunization program with nirsevimab on the prevalence of bronchiolitis cases requiring transfer by a specialized pediatric critical care transport team. Additional objectives included describing and comparing the clinical phenotypes seen in patients presenting with bronchiolitis between the pre-nirsevimab (pre-n) and post-nirsevimab (post-n) periods and comparing the clinical characteristics between immunized and non-immunized patients.

## 2. Materials and Methods

### 2.1. Study Design, Setting and Population

A descriptive and observational study was designed, including patients with bronchiolitis transported by a specialized pediatric transport team from Sant Joan de Déu Hospital between 1 September 2021 and 31 August 2025 (four complete seasons). The study design was prospective, starting from the start of nirsevimab immunization, and included a retrospective review of cases in the two years prior to immunization to allow comparison between the two periods. Patients transported were followed up until their hospital discharge. There were no exclusion criteria. All patients suffered from severe bronchiolitis, and there were no changes in transport policies during the study period.

This study was approved by the institutional Clinical Research Ethics Committee (protocol ID: EOM-01-24) and was performed in compliance with the Declaration of Helsinki.

### 2.2. Data Collection

Demographic, clinical and microbiological data were collected, including age, sex, need for admission to the PICU, and supportive care administered in the PICU (type of respiratory therapy, either non-invasive ventilation (NIV), artificial ventilation (AV) or oxygen therapy alone, as well as the need for inotropes). The length of stay (LOS) in the PICU, the overall length of stay in the hospital, the in-hospital mortality, and immunization with nirsevimab in the previous period were also recorded.

### 2.3. Microbiological Testing

Causative viral agents of bronchiolitis were recorded when available. Samples were collected by performing a nasopharyngeal aspirate or, in intubated patients, tracheal aspirate/bronchoalveolar lavage and tested using a multiplex polymerase chain reaction (PCR) assay for multiple pathogens (including RSV, rhinovirus, metapneumovirus, influenza A and B viruses, parainfluenza virus, adenovirus, bocavirus, seasonal coronaviruses and SARS-CoV-2) either at the referring or receiving hospitals (or both). These tests are commercially available in our country, and all hospitals use them systematically on their patients, so this data is comparable.

Four epidemic seasons were analyzed across two distinct periods: pre- and post-nirsevimab (pre-n, from 1 September 2021 to 31 August 2023, and post-n, from 1 September 2023 to 31 August 2025). Each season was defined as spanning from 1 September to 31 August of the following year. For each season, we assessed the total number of transports of patients with bronchiolitis and the corresponding percentage with respect to the total transports.

All clinical and microbiological variables were compared between the pre-n and post-n periods. Additionally, the clinical characteristics of patients receiving nirsevimab were compared with those of patients who did not receive the intervention.

### 2.4. Statistical Analysis

Data were analyzed using SPSS^®^ version 26.0. Categorical variables were expressed as absolute and relative rates, while continuous variables were expressed as median and interquartile range (IQR). The comparison of categorical variables was performed using the χ2-test, and continuous variables were compared using the Mann–Whitney U-test. The significance level was set at 0.05.

## 3. Results

### 3.1. General Results

A total of 463 episodes of interfacility transports in 459 patients with bronchiolitis were included, with 255 (55.1%) being male. Overall, the median age at admission was 2.5 months (IQR 1.3–5.7). In total, 401 (86.6%) patients required admission to the PICU.

Out of the 463 patients in the sample, 382 (82.5%) required NIV, 58 (12.5%) needed AV, and the remaining needed high-flow oxygen therapy or conventional oxygen cannulas. Only 11 patients (2.4%) needed inotropic support. No patients in the sample required extracorporeal membrane oxygenation (ECMO) therapy, and none died. The median PICU LOS was 4.0 days (IQR 2.0–6.0), and the median in-hospital LOS was 7.0 days (IQR 5.0–9.0).

**Figure 1:**
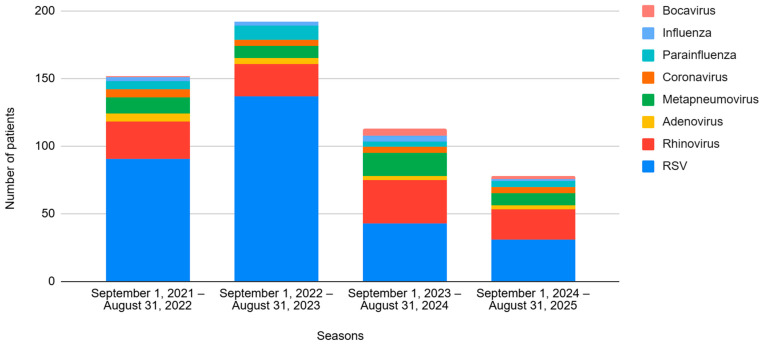
Evolution of the different viruses by season, from 2021 to 2025. Seasons 1 and 2 correspond to the pre-nirsevimab period, and Seasons 3 and 4 correspond to the post-nirsevimab period.

Regarding viral etiology, 302 cases (65.2%) were caused by RSV, followed by rhinovirus (RV) affecting 105 (22.7%) patients. The viral incidences for each season are shown in Figure 1.

A total of 44 patients (9.5%) had bacterial superinfections, 22 patients (4.75%) had bacterial pneumonia, 14 (3.02%) had urinary tract infections, 4 (0.9%) had bacteremia, and 1 (0.2%) had acute otitis media.

The observed immunization coverage in our sample during the post-nirsevimab period was 50% (78/156), an absolute rate markedly lower than that reported in official statistics. However, only 99 patients (28.4%) in the post-nirsevimab period were younger than 6 months of age—the population for whom nirsevimab is recommended—resulting in an adjusted immunization coverage of 78.8% (78/99).

### 3.2. Differences Between the Two Periods: Pre-n vs. Post-n

Patients diagnosed with bronchiolitis were compared between the pre-n and post-n periods as detailed in Table 1. In Supplementary Table S1, the details for each of the four seasons are summarized.

The number of patients suffering from bronchiolitis requiring specialized pediatric transport decreased markedly between the two periods (28.1% to 12.4%; *p* < 0.001).

**Figure 2:**
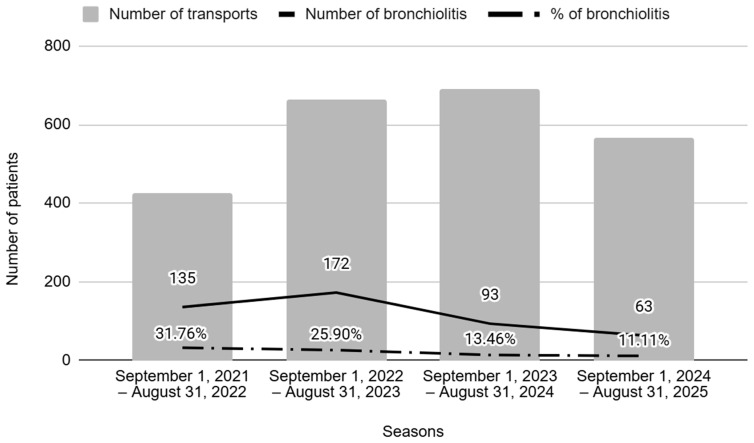
Number of transports and percentage of bronchiolitis per season. Seasons 1 and 2 correspond to the pre-nirsevimab period, and Seasons 3 and 4 correspond to the post-nirsevimab period.

Figure 2 shows the number of episodes along the study timeline, comparing the periods before (seasons 1 and 2) and after (seasons 3 and 4) nirsevimab administration. The results show a significant decrease in the annual number of bronchiolitis cases transported, as well as in their ratio in the post-n period.

Age at admission showed statistically significant differences across the two periods (2.2 vs. 3.4 months, *p* < 0.001). Also, there was a significant decrease in the ratio of children under 6 months between the two periods (81.4% vs. 63.5%, *p* < 0.001).

As for the viral etiology, the incidence of RSV decreased (74.3% vs. 47.4%, *p* < 0.001), while other viruses increased significantly in the post-n period: RV (from 16.6% to 34.6%, *p* < 0.001), metapneumovirus (from 6.8% to 16.7%, *p* = 0.001), and bocavirus (from 0.3% to 3.8%, *p* = 0.007). The number of patients testing positive for two or more viral etiologies (co-infections) was also greater in this period (16.9% vs. 25.0%, *p* = 0.039). No differences were observed in terms of respiratory or hemodynamic support, PICU admission, LOS or mortality.

### 3.3. Differences Between Immunized and Non-Immunized Patients

Comparisons were also made between immunized and non-immunized patients, as shown in Table 2.

There were no differences observed in age or gender between the two groups.

As for the viral etiology, the incidence of RSV was lower in the immunized group (42.3% vs. 69.9%, *p* < 0.001), while RV, metapneumovirus and bocavirus increased significantly (32.1% vs. 20.8%, *p* < 0.030, 24.4% vs. 7.3%, *p* < 0.001, and 7.7% vs. 0.3%, *p* < 0.001, respectively). Also, there were more co-infections in the nirsevimab group (28.2% vs. 17.9%, *p* = 0.037).

There were no differences in the severity of the illness, considering the respiratory and hemodynamic support needed, PICU admission requirement, or mortality.

## 4. Discussion

This study supports the potential of nirsevimab to prevent RSV bronchiolitis while also providing evidence of how this reduction is experienced from a specialized pediatric transport point of view.

The efficacy and safety of nirsevimab have been proven in clinical trials [17,18,19,20,21,22], with extensive literature confirming its impact in reducing both hospitalization and disease severity in infants after its implementation [27,28,29,30,31,32,33,34,35,36,37,38,39,40,41,42,43,44,45,46,47,48]. Accordingly, the present study shows a reduction in the absolute number of children with RSV bronchiolitis requiring specialized transport in the post-n period. Since there have been no changes in hospitalization criteria or transfer requirements for patients with bronchiolitis, we believe that the decrease observed in the number of transfers may be related to nirsevimab vaccination

The observed percentages of bronchiolitis in the post-n seasons were also significantly lower than in the previous seasons, indicating nirsevimab’s effectiveness. Similar findings have been reported across a wide range of clinical settings, including primary care, hospital admissions, and pediatric intensive care units (PICUs), with an estimated effectiveness of around 90% [26,27,28,29,30,31,32,33,34,35,36,37,38,39,40,41,42,43,44,45,46,47,48]. Despite that, the effectiveness at the individual level should be interpreted cautiously, since the immunized and non-immunized groups were not fully comparable, mainly due to the difference in the size of the sample. Potential sources of bias include differences in sample size, the prospective observational and predominantly descriptive study design, the use of a historical cohort for comparison, and the absence of randomization, as all cases managed by a single clinical team were included.

Conversely, no differences were found in terms of clinical course as described by the type and need of respiratory support, the rates of PICU admission, or the combined length of stay. These findings are consistent with the results presented in the meta-analysis by Sumsuzzman DM et al. [48]. However, given the observational nature of the data, causal relationships cannot be established. A possible explanation for this effect could be found in the mechanism of action of nirsevimab; as stated before, this monoclonal antibody targets a glycoprotein of the virus and prevents the entry of RSV into the host cells, neutralizing the virus with a durable effect that provides lasting passive immunity to infants throughout the seasonal period. However, once inside the cell, the disease follows its regular clinical course, regardless of nirsevimab immunization. Moreover, the results could be subject to referral bias, since they only include children requiring specialized transport, potentially excluding those with milder forms of bronchiolitis.

Interestingly, and in line with previous studies [27,36,37,45,46,47], nirsevimab immunization seemed to alter the age profile of transported children, with patients in the post-n seasons being significantly older. This probably corresponds to the immunization strategy designed by the Public Health Commission in Spain, which limited the administration of nirsevimab to infants under six months of age at the beginning of and during the RSV season while also extending its use to children at high risk of severe disease [23].

RSV infection continues to pose a public health burden with relevant costs. In some areas, particularly during the winter season, its incidence can overwhelm hospital resources, with PICU beds being insufficient in some areas to respond to the epidemic. This represents a frequent cause of referral, resulting in infants being transferred hundreds of kilometers away from home [49]. Universal immunization with nirsevimab could be a strategy to mitigate this problem, as derived from data reported in our study. Moreover, nirsevimab could substantially reduce the economic impact of RSV infections. Using a static decision-analytic model, a study by Kieffer A et al. evaluated the health and cost outcomes associated with the use of nirsevimab against the standard of care in the prevention of medically attended RSV-associated lower respiratory tract infections (RSV-MALRTI), resulting in an expected reduction of 290,174 RSV-MALRTI, 24,986 hospitalizations, and expenditures of 612 million US-$ in 2021 [50].

Finally, another hypothesis to discuss is whether the decline of RSV will lead to the emergence of other respiratory viruses. As displayed in our study and in agreement with previous reports [27,37,46,47], there seems to be a shift in viral etiology. The observed incidence of RSV bronchiolitis declined significantly after the introduction of nirsevimab, while the rates of rhinovirus, metapneumovirus, bocavirus and co-infections increased. Although this would suggest a partial viral replacement phenomenon, these pathogens did not reach the epidemiological burden of RSV and were not associated with increased severity in our cohort. Future multicenter studies are needed to determine whether this trend is consistent and clinically relevant.

## 5. Limitations

This study has several limitations. Its observational design limits the ability to draw causal conclusions and leaves room for residual confounding. The retrospective nature of part of the data collection may have led to incomplete or imprecise recording of some variables. In addition, because the cohort mainly included transported patients and a few children with milder bronchiolitis, selection bias cannot be excluded, and the findings may not be fully generalizable. Also, due to the characteristics of the study, the statistical analysis was univariate, which could yield incomplete results. The fact that severity scores were not recorded for all patients and that secondary aspects, such as the need for PICU, respiratory or inotropic support, had to be used, makes the seasons difficult to compare. Finally, the relatively small sample size may have reduced the study’s ability to detect statistically significant differences.

## 6. Conclusions

This study offers data from routine clinical practice on the association between nirsevimab use and a reduction in the number of pediatric patients transferred due to acute bronchiolitis. Although disease severity among transported children remained unchanged, the observed decline in transfers suggests a potential role for nirsevimab in alleviating winter surges in healthcare demand, optimizing pediatric intensive care capacity, and reducing the burden on families and emergency services alike. Taken together, these findings support the potential contribution of passive immunization in infants as a preventive measure against RSV bronchiolitis and add to the growing evidence of its possible public health benefits during the RSV season. Future multicenter studies are needed to validate these findings and evaluate their wider public health and economic impact.

## Figures and Tables

**Table 1:** Comparison between the two periods: Pre-nirsevimab vs. post-nirsevimab.

	Pre-Nirsevimab n = 307	Post-Nirsevimab n = 156	*p*
Total transports, n % Bronchiolitis	1089 28.1	1258 12.4	**<0.001**
Age in months, median (IQR)	2.2 (1.1–4.6)	3.4 (1.7–9.4)	**<0.001**
<6 months, n (%)	250 (81.4)	99 (63.5)	**<0.001**
Sex: males, n (%)	159 (52.0)	95 (60.9)	0.068
**Microbiological data**			
RSV, n (%)	228 (74.3)	74 (47.4)	**<0.001**
Rhinovirus, n (%)	51 (16.6)	54 (34.6)	**<0.001**
Metapneumovirus, n (%)	21 (6.8)	26 (16.7)	**<0.001**
Adenovirus, n (%)	10 (3.3)	6 (3.8)	0.743
Coronavirus, n (%)	11 (3.6)	10 (6.4)	0.167
Parainfluenza, n (%)	16 (5.2)	7 (4.5)	0.734
Influenza, n (%)	6 (2.0)	7 (4.5)	0.119
Bocavirus, n (%)	1 (0.3)	6 (3.8)	**0.007**
Viral coinfection, n (%)	52 (16.9)	39 (25.0)	**0.039**
Bacterial superinfection, n (%) - Pneumonia - UTI - Bacteremia - AOM	27 (8.8) 14 (4.6) 11 (3.6) 2 (0.7) 0 (0)	14 (9.0) 8 (5.1) 3 (1.9) 2 (1.3) 1 (0.6)	0.949
**Supportive therapy and outcomes**			
PICU admission, n (%)	268 (87.3)	133 (85.3)	0.542
Respiratory support			
NIV, n (%)	256 (83.4)	126 (80.8)	0.483
AV, n (%)	37 (12.1)	21 (13.5)	0.665
Inotropic treatment, n (%)	7 (2.3)	4 (2.6)	1.000
ECMO, n (%)	0 (0)	0 (0)	
LOS in hospital, median (IQR)	7.0 (5.0–9.0)	7.0 (5.0–9.0)	0.176
LOS in PICU, median (IQR)	3.0 (2.0–5.5)	4.0 (3.0–6.0)	0.776
Exitus, n (%)	0 (0)	0 (0)	

The two periods were pre-nirsevimab (from 1 September 2021 to 31 August 2023) and post-nirsevimab (from 1 September 2023 to 31 August 2025). RSV: respiratory syncytial virus; UTI: urinary tract infection; AOM: acute otitis media; PICU: pediatric intensive care unit; NIV: non-invasive ventilation; AV: artificial ventilation. ECMO: extracorporeal membrane oxygenation. LOS: length of stay. Note that some patients had multiple viral detections. Data are shown as numbers (percentage) or median (interquartile range). The comparison of categorical variables was performed using the χ2-test, and continuous variables were compared using the Mann–Whitney U-test.

**Table 2:** Comparison between immunized and non-immunized patients.

	Nirsevimab n = 78	No Nirsevimab n = 385	*p*
Age in months, median (IQR)	2.7 (1.4–5.1)	2.4 (1.3–5.8)	0.400
Sex: males, n (%)	45 (57.7)	210 (54.5)	0.610
**Microbiological data**			
RSV, n (%)	33 (42.3)	269 (69.9)	**<0.001**
Rhinovirus, n (%)	25 (32.1)	80 (20.8)	**0.030**
Metapneumovirus, n (%)	19 (24.4)	28 (7.3)	**<0.001**
Adenovirus, n (%)	1 (1.3)	15 (3.9)	0.492
Coronavirus, n (%)	5 (6.4)	16 (4.2)	0.383
Parainfluenza, n (%)	5 (6.4)	18 (4.7)	0.520
Influenza, n (%)	3 (3.8)	10 (2.6)	0.467
Bocavirus	6 (7.7)	1 (0.3)	**<0.001**
Viral coinfection, n (%)	22 (28.2)	69 (17.9)	**0.037**
Bacterial superinfection, n (%) - Pneumonia - UTI - Bacteremia - AOM	6 (7.7) 5 (6.4) 1 (1.3) 0 (0) 0 (0)	35 (9.1) 17 (4.4) 13 (3.4) 4 (1.04) 1 (0.3)	0.692
**Supportive therapy and outcomes**			
PICU admission, n (%)	13 (16.7)	49 (12.7)	0.352
Respiratory support			
NIV, n (%)	59 (75.6)	323 (83.9)	0.080
AV, n (%)	14 (17.9)	44 (11.4)	0.113
Inotropic treatment, n (%)	4 (5.1)	7 (1.8)	0.096
ECMO, n (%)	0 (0)	0 (0)	
LOS in hospital, median (IQR)	7.0 (5.0–9.3)	7.0 (5.0–9.0)	0.658
LOS in PICU, median (IQR)	4.0 (2.8–6.0)	4.0 (2.0–6.0)	0.392
Exitus, n (%)	0 (0)	0 (0)	

IQR: interquartile range. RSV: respiratory syncytial virus; UTI: urinary tract infection; AOM: acute otitis media; PICU: pediatric intensive care unit; NIV: non-invasive ventilation; AV: artificial ventilation. ECMO: extracorporeal membrane oxygenation. LOS: length of stay. Note that some patients had multiple viral detections. Data are shown as numbers (percentage) or median (interquartile range). The comparison of categorical variables was performed using the χ2-test, and continuous variables were compared using the Mann–Whitney U-test.

## Data Availability

The data is stored in an encrypted database and protected by the authors. If anyone needs specific data, they only need to request it from the authors and it will be provided.

## Supplementary Materials

The following supporting information can be downloaded at https://www.mdpi.com/article/10.3390/children13020268/s1, Table S1: Comparison between the four seasons.
