# Supplementary material for: The Impact of Nirsevimab on the Transport of Critically Ill Children
Source: Children (Basel). 2026 Feb 14;13(2):268. doi: 10.3390/children13020268 (PMC12939743; doi:10.3390/children13020268)
Supplement: Supplementary file 1 [file children-13-00268-s001.zip › children-4132081-supplementary.pdf]

Supplementary material

Table S1. Comparison between the four seasons

	**Pre-nirsevimab** n=307		**Post-nirsevimab** n=156		**p**
	**Period 1** n=135 September 1, 2021 – August 31, 2022	**Period 2** n=172 September 1, 2022 – August 31, 2023	**Period 3** n=93 September 1, 2023 – August 31, 2024	**Period 4** n=63 September 1, 2024 – August 31, 2025	
Total transports, n<br>% Bronchiolitis	425<br>31.8	664<br>25.9	691<br>13.5	567<br>11.1	**<0.001**
Age in months, median (IQR)	2.2 (1.1-5.6)	2.1 (1.0-4.3)	2.9 (2.0-9.5)	4.3 (1.4-9.3)	**<0.001**
< 6 months, n (%)	105 (77.8)	145 (84.3)	60 (64.5)	39 (61.9)	**<0.001**
Sex: males, n (%)	67 (49.6)	93 (54.1)	58 (62.4)	37 (58.7)	0.259
**Microbiological data**					
RSV, n (%)	91 (67.4)	137 (79.7)	43 (46.2)	31 (49.2)	**<0.001**
Rhinovirus, n (%)	27 (20.0)	24 (14.0)	32 (34.4)	22 (34.9)	**<0.001**
Metapneumovirus, n (%)	12 (8.9)	9 (5.2)	17 (18.3)	9 (14.3)	**0.005**
Adenovirus, n (%)	6 (4.4)	4 (2.3)	3 (3.2)	3 (4.8)	0.708
Coronavirus, n (%)	6 (4.4)	5 (2.9)	5 (5.4)	5 (7.9)	0.409
Parainfluenza, n (%)	6 (4.4)	10 (5.8)	3 (3.2)	4 (6.3)	0.755
Influenza, n (%)	3 (2.2)	3 (1.7)	5 (5.4)	2 (3.2)	0.367
Bocavirus, n (%)	1 (0.7)	0 (0)	4 (4.3)	2 (3.2)	**0.027**
Viral coinfection, n (%)	21 (15.6)	31 (18.0)	25 (26.9)	14 (22.2)	0.167
Bacterial superinfection,n (%)<br>- Pneumonia<br>- UTI	14 (10.4)<br><br>6 (4.4)<br>7 (5.2)	13 (7.6)<br><br>8 (4.7)<br>4 (2.3)	9 (9.7)<br><br>5 (5.4)<br>2 (2.2)	3 (4.8)<br><br>1 (1.6)<br>1 (1.6)	0.688

- Bacteremia<br>- AOM	1 (0.7)<br>0 (0)	1 (0.6)<br>0 (0)	1 (1.1)<br>1 (1.1)	0 (0)<br>5 (7.9)	
**Supportive therapy and outcomes**					
PICU admission, n (%)	118 (87.4)	150 (87.2)	78 (83.9)	55 (87.3)	0.860
Respiratory support<br>NIV, n (%)<br>AV, n (%)	 112 (83.0)<br>21 (15.6)	 144 (83.7)<br>16 (9.3)	 75 (80.6)<br>10 (10.8)	 51 (81.0)<br>11 (17.5)	 0.914
Inotropic treatment, n (%)	6 (4.4)	1 (0.6)	2 (2.2)	2 (3.2)	0.167
ECMO, n (%)	0 (0)	0 (0)	0 (0)	0 (0)	
LOS in hospital, median (IQR)	7.0 (5.0-9.0)	7.0 (5.0-10.0)	7.0 (5.0-9.0)	7.0 (5.0-9.0)	0.728
LOS in PICU, median (IQR)	3.0 (2.0-6.0)	4.0 (2.0-5.0)	4.0 (3.0-6.0)	4.0 (3.0-7.0)	0.450
Exitus, n (%)	0 (0)		0 (0)		

Periods 1 and 2 were Pre-nirsevimab (from September 1, 2021 to August 31, 2023) periods 3 and 4 were Post-nirsevimab (from September 1, 2023 to August 31, 2025). RSV: Respiratory sincitial virus; UTI: urinary tract infection; AOM: acute otitis media; PICU: Pediatric intensive care unit; NIV: non-invasive ventilation; AV: artificial ventilation. ECMO: extracorporeal membrane oxygenation. LOS: length of stay. Note that some patients had multiple viral detections. Data are shown as number (percentage) or median (interquartile range). The comparison of categorical variables was performed using the $\chi 2$-test, and continuous variables were compared using the Mann–Whitney U-test.
